# Neratinib safety evaluation: real-world adverse event analysis from the FAERS database

**DOI:** 10.3389/fphar.2024.1425171

**Published:** 2024-09-13

**Authors:** Yunhe Fan, Teng Wu, Pengyang Xu, Chuanli Yang, Jie An, Haijia Zhang, Mureed Abbas, Xiushan Dong

**Affiliations:** ^1^ Department of General Surgery, Shanxi Bethune Hospital, Shanxi Academy of Medical Sciences, Third Hospital of Shanxi Medical University, Tongji Shanxi Hospital, Taiyuan, China; ^2^ Third Hospital of Shanxi Medical University, Shanxi Bethune Hospital, Shanxi Academy of Medical Sciences, Tongji Shanxi Hospital, Taiyuan, China; ^3^ Key Laboratory of Environmental Medical Engineering and Education Ministry, School of Public Health, Southeast University, Nanjing, Jiangsu, China; ^4^ Department of Preventive Medicine, School of Public Health, Southeast University, Nanjing, China; ^5^ Modern Research Center for Traditional Chinese Medicine, the Key Laboratory of Chemical Biology and Molecular Engineering of Ministry of Education, Shanxi University, Taiyuan, Shanxi, China

**Keywords:** neratinib, FAERS, adverse drug events, disproportionality methods, safety signal

## Abstract

**Aims:**

Neratinib has emerged as significant theraputic option for breast cancer treatment. However, despite its approval, numerous adverse drug events (ADEs) associated to it remain unrecognized and unreported. This study aims to mine and analyze the signals of ADEs related to neratinib from the US Food and Drug Administration Adverse Event Reporting System (FAERS) database, providing insights for safe and rational clinical use of drug.

**Methods:**

All the neratinib-related ADEs data were collected from FAERS database from the third quarter (Q3) of 2017 to the fourth quarter (Q4) of 2023. After standardizing the data, 4 disproportionality methods were used to assess the correlation between neratinib and ADEs.

**Results:**

Of the 1,544 ADEs implicating neratinib as the primary suspected drug, a combined total of 48 preferred terms (PTs) and 10 system organ classes (SOCs) showed significant disproportionality accross all four algorithms simultaneously. These SOCs included gastrointestinal disorders (n = 2,564, ROR 7.14), general disorders and administration site conditions (n = 958, ROR 0.77) and injury poisoning and procedural complications (n = 474, ROR 0.58) among others. Upon comparison with the neratinib manual, 34 ADEs not documented in the manual were found at the PT level.

**Conclusion:**

Our study provide new real-world evidence for drug safety information of neratinib. While the majority of our findings were aligned with the information provided in the manual. We identified additional ADEs not previously documented. Consequently, further studies are needed to validate unreported ADEs to ensure the efficacy and safety of neratinib for patients.

## 1 Introduction

Breast cancer is the primary cause of cancer-related-deaths among women worldwide. As of 2020, the standardized mortality rate for breast cancer globally stood at 13.6 per 100,000 individuals (Sung et al., 2021). This disease has had a significant impact on public health, with its incidence and mortality rates steadily rising over the years. China, in particular, has witnessed a consistent increase in both the incidence and fatality rates of female breast cancer from 1990 to 2019 (Malmgren et al., 2023), which seriously jeopardizing the physical and mental health of women and imposing substantial burdens on families and society. Thus, ensuring effective and safe pharmacological treatments is crucial in this context.

Human epidermal growth factor receptor-2 (HER2) encoded by oncogene ErbB2, exhibits amplification and over-expression in 20%–30% of breast cancer cases (Gandhi and Das, 2019). The positive expression of HER2 is associated with the poor prognosis of breast cancer (Slamon et al., 2001), which is known for its aggressive nature. Therefore, effective anti-HER2-targeted therapies are crucial for improving the prognosis of patients (Wahdan-Alaswad et al., 2020). According to guidelines, Trastuzumab, pertuzumab, and taxane for first-line treatment and trastuzumab deruxtecan for second-line treatment are recommended. Not least, other HER2-selective tyrosine kinase inhibitors (tucatinib and neratinib) are treatment options for third-line and beyond (Giordano et al., 2022; Morgovan et al., 2024). Neratinib, an oral pan HER inhibitor, plays a significant role in this regard by irreversibly inhibiting the tyrosine Kinase activity of HER1, HER2 and HER4. This leads to reduced autophosphorylation and downstream signaling, ultimately inhibiting cell growth. (Tiwari et al., 2016; Guo et al., 2023; Deeks, 2017). Neratinib received approval from the US Food and Drug Administration (FDA) in July 2017 and was subsequently approved for marketing in China in April 2020. According to the 2021 Chinese Guidelines and Norms for the Diagnosis and Treatment of Breast Cancer, Patients with HER2-positive breast cancer who have completed trastuzumab treatment and are at risk of recurrence may be considered for 1 year of neratinib intensive therapy, Here is a detailed instruction of neratinib on the FDA website (https://www.accessdata.fda.gov/drugsatfda_docs/label/2017/208051s000lbl.pdf).

However, like any medication, neratinib usage entails the risk of adverse reactions. Given the relatively short period since its introduction to the market, safety data for neratinib stem from clinical trial studies, and there is no systematic study of adverse event signals based on big data after marketing. The FDA Adverse Event Reporting System (FDA Adverse Event Reporting System, FAERS) is a database designed to support the FDA’s post-marketing surveillance program for drugs and therapeutic biologics that includes all adverse event information and medication error information collected by the FDA (Yunusa et al., 2022). Data from FAERS are used to evaluate the safety and efficacy of drugs. In this paper, neratinib-related data was mined from FAERS, evaluate the data using various signal quantification techniques from different perspectives, and alert about the potential adverse reactions, so as to avoid clinical drug risks and ensure the safety of patients.

## 2 Methods

### 2.1 Data source

The ADEs data used in this study were obtained from the FAERS database, which has been publicly accessible since 2004 for collecting adverse event reports from various sources including healthcare professionals, pharmaceutical manufacturers and patients (Zhou and Hultgren, 2020). To investigate ADEs linked to neratinib, data spanningfrom the period from the third quarter (Q3) of year 2017 to the fourth quarter (Q4) of year 2023 were rerieved from the FAERS database. Subsequently, the data were imported into MySQL 15.0 and processed using Navicat Premium 15 software, facilitating comprehensive analysis (Brown, 2004).

### 2.2 Data extraction and analysis

In this study, neratinib was employed as the suspected drug, and its name was coded using Medex_UIMA_1.8.3. The data obtained from the FAERS database were pre-processed using Statistical Analysis System (SAS) and MySQL.n. To ensure the integrity of the data. Duplicate reports with identical cases in the DEMO table were removed. Furthermore, the recent version of the Medical Dictionary of Regulatory Activities version (MedDRA 25.0) was utilized to match the preferred terms (PTs) for ADEs associated with neratinib alongwith the corresponding system organ classes (SOCs). Clinical characteristics such as age, gender, reporter, reporting area, reporting time, and patient outcomes for those experiencing neratinib-related adverse events were collected.

### 2.3 Data mining algorithm

To identify the potential association between neratinib and ADEs a disproportionality analysis was conducted. This analysis is considered a critical analytical tool in pharmaco-vigilance, aiming to assess the correlation between drugs and ADEs by comparing the ratio of observed frequencies in exposed and non-exposed populations using 2 × 2 contingency tables (Table1). In this study, four disproportionality methods were simultaneously employed to detect drug ADE signals: reporting odds ratios (ROR) (Rothman et al., 2004), proportional reporting ratios (PRR) (Evans et al., 2001), Bayesian Confidence Propagation Neural Network (BCPNN) (Bate et al., 1998), and Empirical Bayesian Geometric Mean (EBGM) techniques (DuMouchel, 1999). The advantage of ROR is that it can correct the bias caused by the small number of reports for certain events. The advantage of PRR is its higher specificity compared to ROR. BCPNN excels in integrating multi-source data and performing cross-validation. The advantage of MGPS is its ability to detect signals from rare events. The formulas and cut-off thresholds of the four algorithms are shown in Supplementary Table S1, and statistical analyses were performed using R software. Higher values indicated stronger signal strength, suggesting a more robust association between the target drug and the ADE.

**TABLE 1:** Basic information on Adverse Drug Events (ADEs) related to neratinib from the FAERS database.

Variable	Number of events (%)
Year	
2017	56 (3.63)
2018	765 (49.55)
2019	190 (12.31)
2020	193 (12.50)
2021	153 (9.91)
2022	84 (5.44)
2023	103 (6.67)
Sex	
Female	50 (3.24)
Male	2 (0.13)
Unkown	1492 (96.63)
Age (years)	
18–45	10 (0.65)
45–65	28 (1.81)
65–75	2 (0.13)
≥75	3 (0.19)
Unknow	1501 (97.22)
Reporter	
Pharmacist	773 (50.06)
Other health-professional	447 (28.95)
Consumer	199 (12.89)
Physician	115 (7.45)
Lawyer	3 (0.19)
Unkown	7 (0.45)
Reported countries	
United States	1321 (85.56)
Argentina	51 (3.30)
Other	172 (11.14)
Outcomes	
hospitalization	315 (37.28)
Death	191 (22.60)
life threatening	10 (1.18)
Disability	4 (0.47)
required intervention to Prevent Permanent Impairment/Damage	1 (0.12)
other serious	324 (38.34)
time to onset (days)	
<7	185 (14.27)
7–28	74 (5.71)
28–60	41 (3.16)
≥60	58 (4.48)
Unknow	938 (72.38)

**FIGURE 1:**
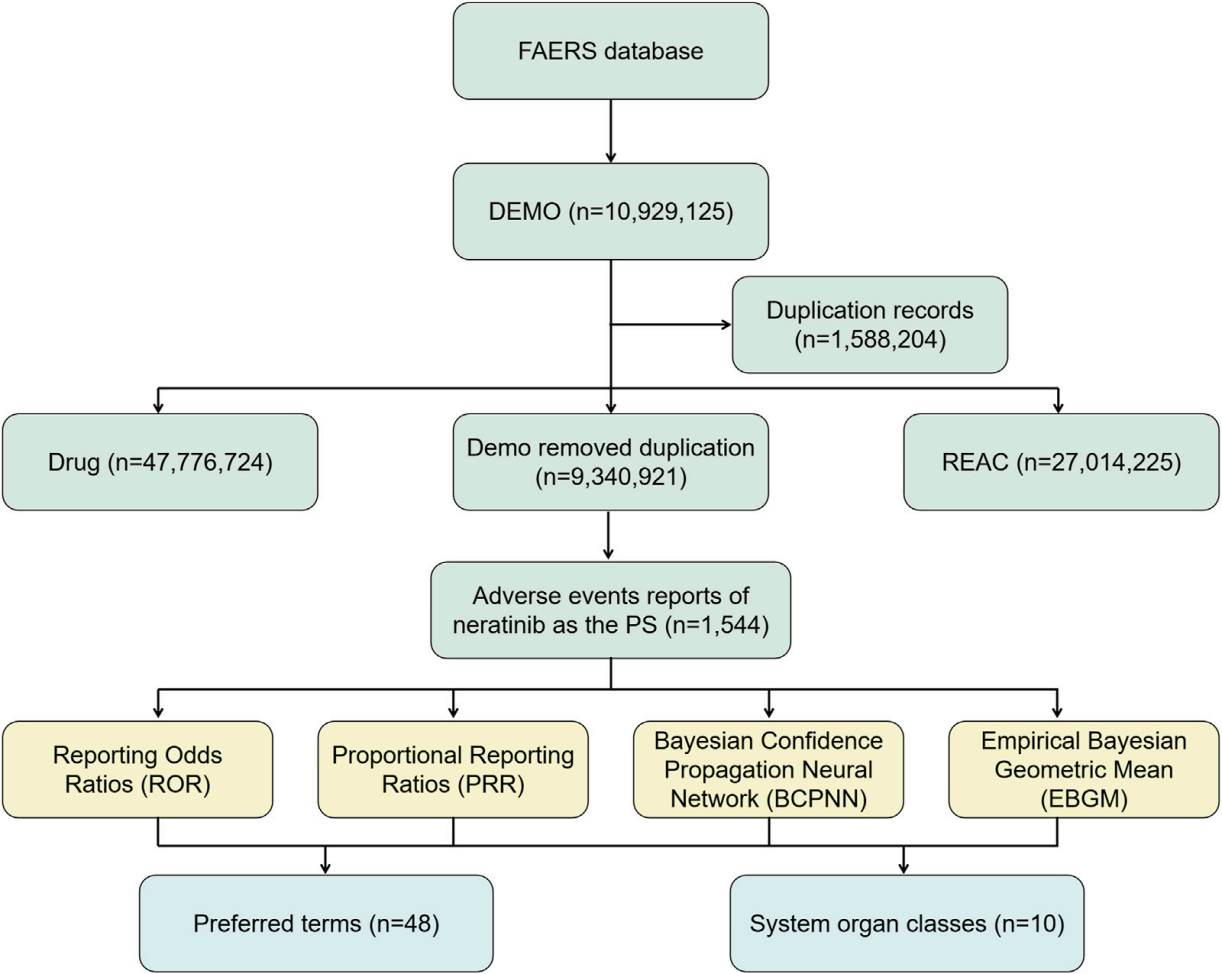
The flow diagram of selecting and analyzing neratinib-related ADEs from FAERS database.

Effective ADE results should meet the positive signal selection criteria of all four algorithms mentioned above. All data related to neratinibunderwent processing and statistical analysis using Navicat Premium 15 software. The general flow chart of this study is illustrated in Figure 1.

## 3 Results

### 3.1 Basic information about adverse events of neratinib

From the period spanning from third quarter of 2017 to the fourth quarter of 2023, a total of 10,929,125 adverse event reports from the FAERS database. Following the removal of duplicate data and exclusion of ADEs that could not be evaluated, a total of 1,544 adverse event reports implicating neratinib as the primary suspected drug were extracted, involving 48 PTs and a total of 22 SOCs.Notably, the available data showed that female patients significantly outnumbered male patients (3.24%/0.13%). However, due to the high number of reports (>90%) of missing gender and age information in the database, this information still needs to be further verified. Regarding age, a significant proportion of data (>90%) did not provide age information, which hindered our ability to thoroughly understand the association between age and adverse events. However, among reports with clear age data, the most common age group was 45–65 years old. Notably, the majority (50.06%) of reports were provided by pharmacists. The vast majority of reports originated from the United States, accounting for 85.56% of the total. In terms of clinical outcomes, except for unknown serious medical events (38.34%), hospitalization emerged as the most frequently reported serious adverse event, accounting for 37.28% of cases, with a total of 315 cases. Death and life-threatening events followed closely with 191 (22.60%) and 10 (1.18%) cases, respectively. Details can be found in Table 1.

### 3.2 Signals detection based on system organ class levels

**FIGURE 2:**
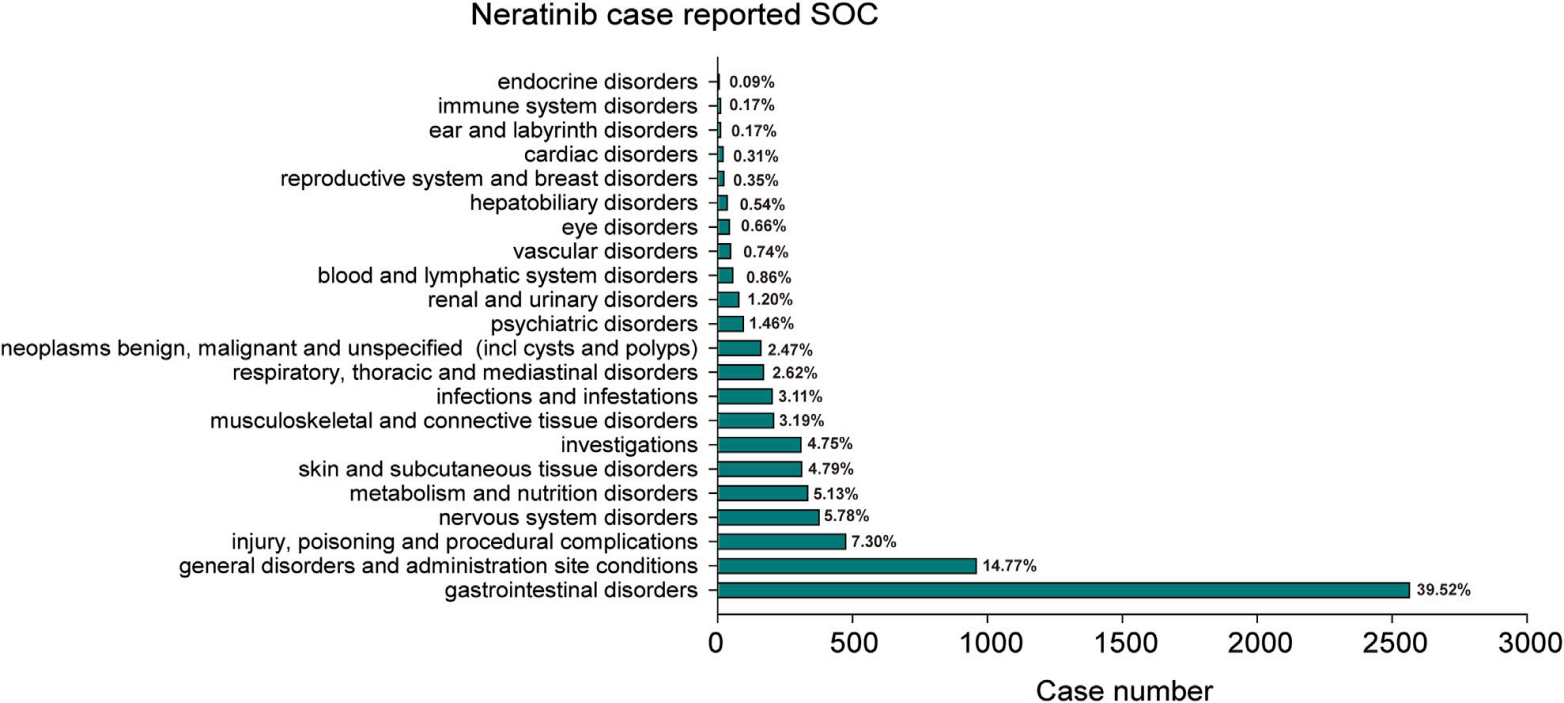
The bar plot depicts a statistical graph showing the distribution of 22 SOCs associated with case reports of neratinib adverse events. The percentage values labeled in the figure represent the proportion of cases where neratinib resulted in an adverse event within each SOC.

The statistical analysis showed that adverse reactions related to neratinib involved 22 SOCs (Figure 2). Among these, the top three in terms of total ADE cases under each SOC were gastrointestinal disorders (2,564, 39.52%), general disorders and administration site conditions (958, 14.77%) and injury poisoning and procedural complications (474, 7.31%). Notably, gastrointestinal disorders (n = 2,564, ROR 7.14, PRR 4.71, IC 2.24, EBGM 4.71) exhibited strongly positive signals across all four algorithms (Table 2). The outcome aligns consistently with the SOC corresponding to common adverse reactions listed in the drug label, indicate a high level of confidence in the data.

**TABLE 2:** The signal strength of Adverse Drug Events (ADEs) of neratinib at the SOC level in FAERS database.

SOC	Case reports	ROR (95% CI)	PRR (95% CI)	Chisq	BCPNN (IC025)	EBGM (EBGM05)
gastrointestinal disorders	2564	7.14 (6.79–7.5)	4.71 (4.53–4.9)	8181.8	2.24 (2.17)	4.71 (4.52)
metabolism and nutrition disorders	333	2.58 (2.31–2.88)	2.5 (2.27–2.76)	305.94	1.32 (1.16)	2.5 (2.28)
Investigations	308	0.77 (0.69–0.87)	0.78 (0.69–0.88)	19.6	−0.35 (−0.52)	0.78 (0.71)
skin and subcutaneous tissue disorders	311	0.77 (0.69–0.87)	0.78 (0.69–0.88)	19.88	−0.35 (−0.52)	0.78 (0.71)
general disorders and administration site conditions	958	0.77 (0.72–0.83)	0.8 (0.75–0.85)	55.87	−0.31 (−0.41)	0.8 (0.76)
nervous system disorders	375	0.71 (0.64–0.79)	0.73 (0.66–0.81)	40.96	−0.46 (−0.61)	0.73 (0.67)
neoplasms benign, malignant and unspecified (incl cysts and polyps)	160	0.68 (0.58–0.79)	0.69 (0.59–0.81)	23.78	−0.54 (−0.77)	0.69 (0.6)
hepatobiliary disorders	35	0.62 (0.45–0.87)	0.63 (0.45–0.88)	7.91	−0.68 (−1.15)	0.63 (0.47)
injury, poisoning and procedural complications	474	0.58 (0.53–0.64)	0.61 (0.56–0.66)	129.64	−0.7 (−0.84)	0.62 (0.57)
musculoskeletal and connective tissue disorders	207	0.58 (0.51–0.67)	0.6 (0.52–0.69)	59.34	−0.74 (−0.94)	0.6 (0.53)
renal and urinary disorders	78	0.55 (0.44–0.69)	0.56 (0.45–0.69)	28.02	−0.84 (−1.16)	0.56 (0.46)
respiratory, thoracic and mediastinal disorders	170	0.54 (0.46–0.63)	0.55 (0.47–0.64)	65.15	−0.86 (−1.08)	0.55 (0.49)
infections and infestations	202	0.53 (0.46–0.61)	0.55 (0.48–0.63)	80.56	−0.87 (−1.07)	0.55 (0.49)
reproductive system and breast disorders	23	0.52 (0.34–0.78)	0.52 (0.34–0.78)	10.24	−0.94 (−1.52)	0.52 (0.37)
blood and lymphatic system disorders	56	0.49 (0.38–0.64)	0.49 (0.38–0.63)	29.45	−1.02 (−1.39)	0.49 (0.4)
ear and labyrinth disorders	11	0.38 (0.21–0.69)	0.38 (0.21–0.68)	10.99	−1.39 (−2.2)	0.38 (0.23)
vascular disorders	48	0.37 (0.28–0.49)	0.37 (0.28–0.49)	51.18	−1.42 (−1.82)	0.37 (0.3)
endocrine disorders	6	0.34 (0.15–0.76)	0.34 (0.15–0.76)	7.64	−1.55 (−2.62)	0.34 (0.17)
eye disorders	43	0.33 (0.24–0.44)	0.33 (0.25–0.44)	59.24	−1.59 (−2.02)	0.33 (0.26)
psychiatric disorders	95	0.25 (0.2–0.3)	0.26 (0.21–0.32)	212.5	−1.95 (−2.24)	0.26 (0.22)
cardiac disorders	20	0.14 (0.09–0.22)	0.14 (0.09–0.22)	103.6	−2.79 (−3.41)	0.14 (0.1)
immune system disorders	11	0.13 (0.07–0.24)	0.13 (0.07–0.23)	63.7	−2.92 (−3.74)	0.13 (0.08)

### 3.3 Signals detection based on preferred term levels

**FIGURE 3:**
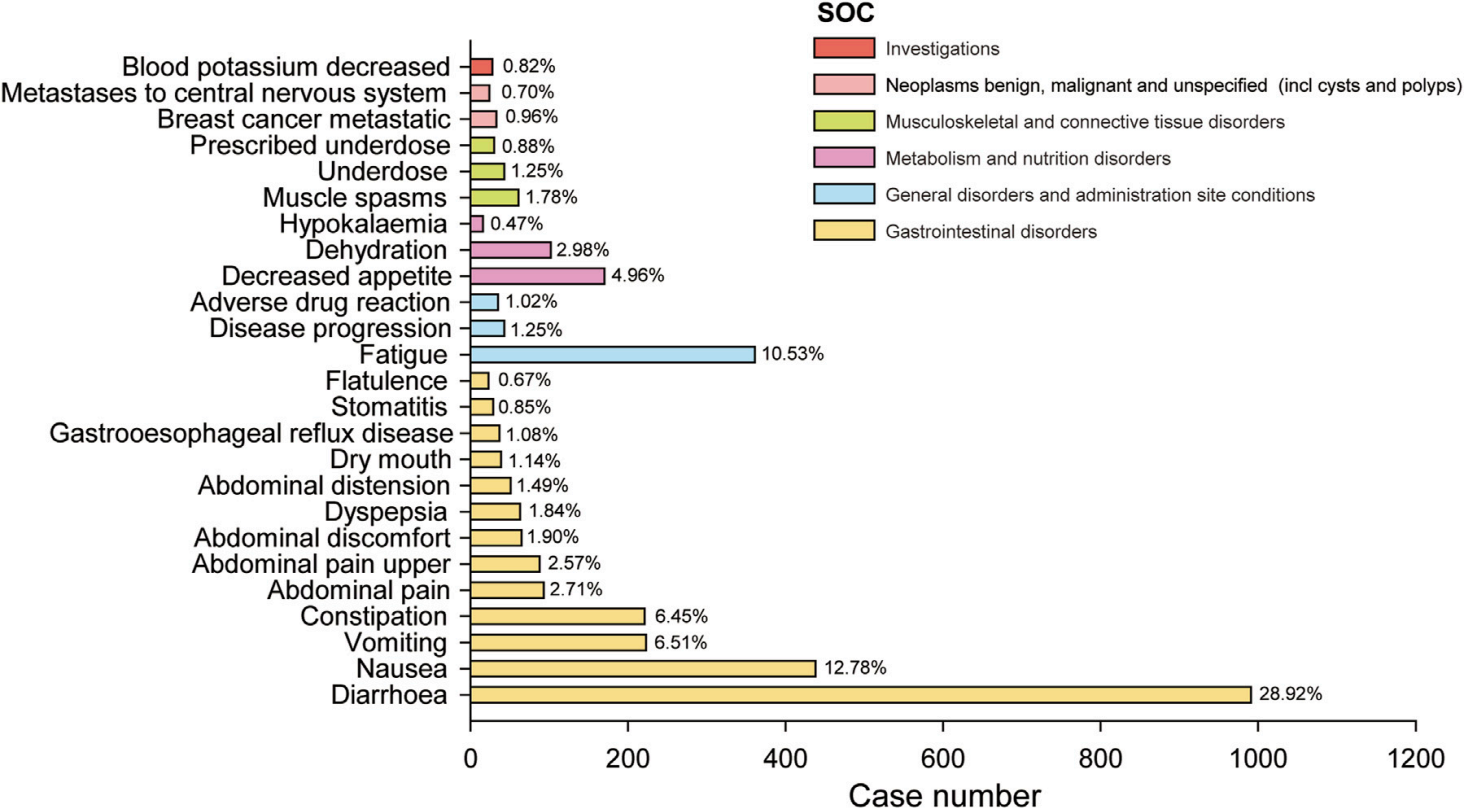
The bar blot illustrates the top 25 adverse event signal strengths associated with neratinib in the FDA Adverse Event Reporting System, sorted by preferred term level for IC025. Each color represents the System Organ Class (SOC) corresponding to the Preferred Terms (PT). The percentages indicate the number of case reports under that PT relative to the sum of the top 25 case reports in IC025.

We further examined PT signals and identified a total of 48 significant disproportionality PTs across all four algorithms simultaneously. Notably, the BCPNN approach showed greater caution, focusing specificity and reducing the risk of misclassification. We ranked 48 PTs based on their signal intensity (IC025 value) in descending order and narrowing our focus to the top 25 PTs, we categorized them into their respective System Organ Classes (SOCs). Among these top 25 PTs, breast cancer metastatic displayed the highest IC025 signal intensity (IC025 = 5.05), while diarrhoea had the highest recorded case number (n = 991). Moreover, several additional adverse reactions were also included, such as early satiety, bladder spasm, metastases to central nervous system, onycholysis, onychoclasis, faeces pale and diarrhoea (Figure 3). We ranked the 48 PTs in descending order based on the number of case reports (Table 3). Among them, the top 5 preferred terms (PTs) with the highest number of reported cases were diarrhoea (n = 991), nausea (n = 438), fatigue (n = 361), vomiting (n = 223) and constipation (n = 221). In addition to the side effects alreadydocumented in the instructions, this study also identified 34 ADEs that were not previously included in the instructions.

**TABLE 3:** The PTs of neratinib ranked by the number of case report in FAERS database.

PT	SOC	Case reports	ROR (95% CI)	PRR (95% CI)	Chisq	BCPNN(IC025)	EBGM (EBGM05)
Diarrhoea	gastrointestinal disorders	991	15.92 (14.88–17.04)	13.64 (12.86–14.47)	11,703.35	3.77 (3.67)	13.6 (12.85)
Nausea	gastrointestinal disorders	438	5.8 (5.26–6.39)	5.48 (4.97–6.04)	1,620.94	2.45 (2.31)	5.47 (5.04)
Fatigue	general disorders and administration site conditions	361	4.25 (3.82–4.73)	4.07 (3.69–4.49)	847.05	2.02 (1.87)	4.07 (3.72)
Vomiting	gastrointestinal disorders	223	5 (4.38–5.72)	4.87 (4.25–5.59)	688.95	2.28 (2.09)	4.86 (4.35)
Constipation^a^	gastrointestinal disorders	221	9.83 (8.59–11.24)	9.53 (8.31–10.93)	1688.92	3.25 (3.06)	9.51 (8.5)
decreased appetite	metabolism and nutrition disorders	170	6.8 (5.84–7.92)	6.65 (5.68–7.78)	817.29	2.73 (2.51)	6.64 (5.84)
Dehydration	metabolism and nutrition disorders	102	8.48 (6.98–10.32)	8.37 (6.88–10.18)	661.46	3.06 (2.78)	8.35 (7.09)
abdominal pain	gastrointestinal disorders	93	4.05 (3.3–4.97)	4 (3.29–4.87)	210.23	2 (1.71)	4 (3.37)
abdominal pain upper^a^	gastrointestinal disorders	88	4.19 (3.39–5.17)	4.14 (3.34–5.14)	210.42	2.05 (1.75)	4.14 (3.47)
abdominal discomfort^a^	gastrointestinal disorders	65	3.26 (2.55–4.16)	3.24 (2.56–4.1)	100.84	1.69 (1.34)	3.24 (2.64)
Dyspepsia	gastrointestinal disorders	63	6.73 (5.25–8.63)	6.68 (5.18–8.62)	304.1	2.74 (2.38)	6.67 (5.42)
muscle spasms	musculoskeletal and connective tissue disorders	61	3.34 (2.6–4.3)	3.32 (2.57–4.28)	99.07	1.73 (1.37)	3.32 (2.69)
abdominal distension	gastrointestinal disorders	51	4.96 (3.76–6.53)	4.92 (3.74–6.47)	159.55	2.3 (1.9)	4.92 (3.91)
disease progression^a^	general disorders and administration site conditions	43	3.41 (2.53–4.61)	3.4 (2.53–4.56)	72.78	1.76 (1.34)	3.39 (2.64)
Underdose^a^	injury, poisoning and procedural complications	43	4.7 (3.48–6.34)	4.67 (3.48–6.27)	124.18	2.22 (1.8)	4.67 (3.63)
dry mouth	gastrointestinal disorders	39	5.18 (3.78–7.1)	5.16 (3.77–7.06)	130.7	2.37 (1.92)	5.15 (3.96)
gastrooesophageal reflux disease^a^	gastrointestinal disorders	37	4.68 (3.39–6.47)	4.66 (3.41–6.38)	106.51	2.22 (1.76)	4.66 (3.56)
adverse drug reaction^a^	general disorders and administration site conditions	35	3.22 (2.31–4.49)	3.21 (2.3–4.48)	53.27	1.68 (1.21)	3.21 (2.43)
breast cancer metastatic^a^	neoplasms benign, malignant and unspecified (incl cysts and polyps)	33	33.46 (23.7–47.17)	33.29 (23.86–46.45)	1025.66	5.05 (4.56)	33.04 (24.79)
prescribed underdose^a^	injury, poisoning and procedural complications	30	9.67 (6.75–13.84)	9.63 (6.77–13.7)	231.46	3.26 (2.75)	9.61 (7.11)
Stomatitis	gastrointestinal disorders	29	4.22 (2.93–6.08)	4.21 (2.9–6.11)	70.95	2.07 (1.55)	4.21 (3.1)
blood potassium decreased^a^	Investigations	28	9.46 (6.52–13.71)	9.42 (6.49–13.67)	210.32	3.23 (2.71)	9.4 (6.89)
metastases to central nervous system^a^	neoplasms benign, malignant and unspecified (incl cysts and polyps)	24	17.22 (11.52–25.73)	17.16 (11.6–25.4)	363.77	4.1 (3.53)	17.09 (12.21)
Flatulence^a^	gastrointestinal disorders	23	4.18 (2.77–6.29)	4.16 (2.76–6.28)	55.3	2.06 (1.48)	4.16 (2.95)
Hypokalaemia^a^	metabolism and nutrition disorders	16	3.38 (2.07–5.52)	3.37 (2.06–5.5)	26.71	1.75 (1.07)	3.37 (2.24)
Onychoclasis^a^	skin and subcutaneous tissue disorders	13	15.3 (8.87–26.39)	15.27 (8.82–26.44)	172.76	3.93 (3.17)	15.22 (9.64)
skin fissures	skin and subcutaneous tissue disorders	13	6.1 (3.54–10.51)	6.09 (3.52–10.54)	55.18	2.6 (1.85)	6.08 (3.85)
dermatitis acneiform^a^	skin and subcutaneous tissue disorders	8	13.05 (6.52–26.14)	13.04 (6.57–25.89)	88.65	3.7 (2.76)	13 (7.27)
gastrointestinal sounds abnormal^a^	gastrointestinal disorders	7	13.73 (6.54–28.85)	13.72 (6.51–28.89)	82.28	3.77 (2.77)	13.68 (7.35)
faeces soft^a^	gastrointestinal disorders	6	6.16 (2.76–13.72)	6.16 (2.76–13.76)	25.87	2.62 (1.55)	6.15 (3.14)
nail disorder	skin and subcutaneous tissue disorders	6	6.96 (3.13–15.52)	6.96 (3.12–15.55)	30.56	2.8 (1.73)	6.95 (3.55)
breast cancer stage iv^a^	neoplasms benign, malignant and unspecified (incl cysts and polyps)	6	9.88 (4.43–22.02)	9.87 (4.42–22.05)	47.73	3.3 (2.23)	9.85 (5.04)
metastases to lung^a^	neoplasms benign, malignant and unspecified (incl cysts and polyps)	6	4.67 (2.1–10.41)	4.67 (2.09–10.43)	17.28	2.22 (1.15)	4.66 (2.39)
nasal dryness^a^	respiratory, thoracic and mediastinal disorders	6	9.58 (4.3–21.36)	9.58 (4.29–21.4)	45.98	3.26 (2.19)	9.56 (4.89)
brain neoplasm^a^	neoplasms benign, malignant and unspecified (incl cysts and polyps)	5	4.98 (2.07–11.97)	4.98 (2.06–12.03)	15.87	2.31 (1.16)	4.97 (2.39)
blood magnesium decreased^a^	Investigations	5	5.15 (2.14–12.38)	5.15 (2.13–12.44)	16.68	2.36 (1.21)	5.14 (2.47)
Enteritis^a^	gastrointestinal disorders	4	5.23 (1.96–13.96)	5.23 (1.96–13.94)	13.67	2.39 (1.12)	5.23 (2.3)
early satiety^a^	general disorders and administration site conditions	4	29.42 (11–78.69)	29.4 (11.03–78.34)	108.99	4.87 (3.6)	29.21 (12.82)
Paronychia^a^	infections and infestations	4	8.15 (3.05–21.73)	8.14 (3.06–21.69)	25.01	3.02 (1.76)	8.13 (3.58)
faeces pale^a^	gastrointestinal disorders	3	14.37 (4.62–44.66)	14.36 (4.61–44.76)	37.17	3.84 (2.42)	14.32 (5.54)
gastrointestinal toxicity^a^	gastrointestinal disorders	3	5.71 (1.84–17.73)	5.71 (1.83–17.8)	11.64	2.51 (1.1)	5.7 (2.21)
Faecaloma^a^	gastrointestinal disorders	3	5.54 (1.78–17.18)	5.53 (1.77–17.24)	11.13	2.47 (1.05)	5.53 (2.14)
Onycholysis^a^	skin and subcutaneous tissue disorders	3	16.05 (5.16–49.89)	16.04 (5.15–49.99)	42.16	4 (2.58)	15.99 (6.19)
nail discolouration^a^	skin and subcutaneous tissue disorders	3	7.01 (2.26–21.75)	7 (2.25–21.82)	15.42	2.81 (1.39)	6.99 (2.71)
metastatic neoplasm^a^	neoplasms benign, malignant and unspecified (incl cysts and polyps)	3	7.68 (2.47–23.83)	7.67 (2.46–23.91)	17.38	2.94 (1.52)	7.66 (2.97)
wound haemorrhage^a^	injury, poisoning and procedural complications	3	6.9 (2.22–21.43)	6.9 (2.21–21.51)	15.11	2.78 (1.37)	6.89 (2.67)
breast cellulitis^a^	infections and infestations	3	106.74 (33.92–335.84)	106.69 (34.23–332.53)	306.24	6.7 (5.26)	104.04 (39.87)
bladder spasm^a^	renal and urinary disorders	3	19.54 (6.28–60.77)	19.53 (6.27–60.87)	52.51	4.28 (2.86)	19.45 (7.53)

^a^ stands for ADEs, that is not recorded in the specification.

### 3.4 Time scans of safety signals

**FIGURE 4:**
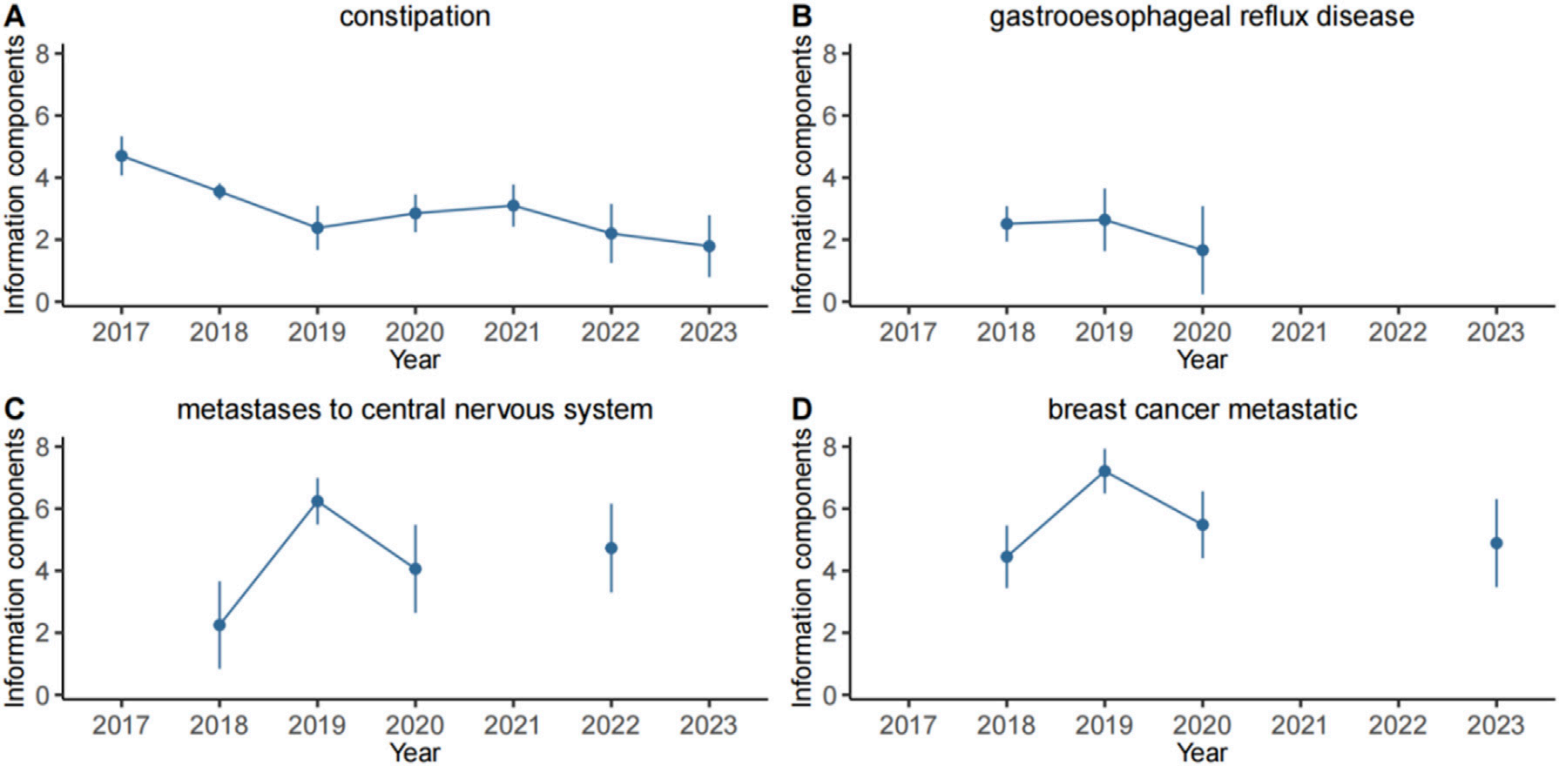
Information component and its 95% credibility interval over time for neratinib associated adverse events. **(A)** constipation, **(B)** gastrooesophageal reflux disease, **(C)** metastases to central nervous system, **(D)** breast cancer metastatic. The error bars show the 95% credibility interval (CI) of the information component (IC), a steady upward trend in the IC curve with narrowed 95% CI indicates a stable signal and a strong association. The credibility interval (CI) of the information component (IC) decreased over time as more data is accumulated, resulting in a smaller confidence interval. When the value 0 is excluded from the CI, a signal is flagged.

In this study, four specific PTs: constipation, gastrooesophageal reflux disease, breast cancer metastatic and metastases to central nervous system which were not mentioned in the instructions. Their IC025 values were analyzed in correlation with neratinib (Figure 4). The findings revealed that IC025 value for constipation was consistantly higher than 0 from 2017 to 2023, indicating a strong correlation with neratinib. Similarly, the IC025 values for the other 3 PTs remained above 0 from 2017 to 2020. Moreover, the IC025 value of metastases to central nervous system in 2022 were greater than 0, and the IC025 value of breast cancer metastatic in 2023 were greater than 0. Taken together, these observations suggest a notable correlation between neratinib and these 3 PTs as well.

## 4 Discussion

In this study, ADEs data was extracted from FAERS database, and 4 disproportionality methods were employed to mine and analyzed signals related to neratinib associated ADEs. The results showed that ADE signals were mainly concentrated in gastrointestinal disorders, general disorders and administration site conditions, metabolism and nutrition disorders, etc., which were basically consistent with the contents outlined in the manual, This consistancy confirms the validity and reliability of the study methodology.

### 4.1 Demographic characteristics of ADEs to neratinib

The adverse event reports associated with neratinib collected in this study indicated a significant gender dicrepancy, with female patients comprising a significantly higher proportion (96.15%) compared to male patients (3.85%) when we exclude the patients with unknown gender, due to the specific indications of neratinib was breast cancer (Chan et al., 2021). Regarding the age composition, although a considerable portion of the data lacking specific age details, among patients with known age, three-quarters of ADEs occurred in middle-aged and elderly patients aged over 45 years. According to the data reported by countries, the United States accounted for the highest number of reported cases, representing 85.56% of the total reports. This indicates that the United States attaches great importance to drug safety and also warns other countries to improve the monitoring and reporting of adverse drug reactions. After neratinib received approval in 2017, the number of ADEs in 2018 reached 765, indicating that the drug was highly valued at its initial use. However, the significant reduction in ADEs by 2022 was mainly due to the COVID-19 pandemic, which severely impacted people’s lives.

### 4.2 ADEs involve systematic organ classification characteristics

Our findings revealed a total of 48 ADE signals associated with neratinib, involving 10 systematic organ classifications, among which the ADE cases of gastrointestinal disorders were the most frequent, followed by general disorders and administration site conditions, metabolic and nutritional diseases. Gastrointestinal disorders were the most frequently reported adverse events recorded in the manual. This study also detected constipation, gastrooesophageal reflux disease, gastrointestinal sounds abnormal, enteritis and other rare ADE in the gastrointestinal tract, suggesting that if such diseases are found in the course of treatment, the possibility of drug-induced correlation should be considered. Fatigue is the most commonly reported adverse event in general disorder and administration site conditions, followed by disease progression and adverse drug reaction. Neratinib being a targeted small molecule drug, is exclusively administered to cancer patients, which is speculated to be caused by tumor progression in some patients and the primary disease is not controlled. Signals such as Dehydration, decreased appetite, hypokalemia and other were detected within metabolic and nutritional disorders, suggesting that clinical attention should be paid to patients’ vital signs and blood indicators during medication. In another study, ADEs of trastuzumab deruxtecan (T-DXd) and trastuzumab emtansine (T-DM1) were described and compared with neratinib, showing that T-DXd and T-DM1 exhibit higher probability of ADEs from many SOCs than neratinib (such as hepatobiliary disorders, nervous system disorders, blood and lymphatic disorders etc.). This indicates that neratinib may be more secure than T-DXd and T-DM1.

### 4.3 New ADEs of neratinib

A total of 34 ADEs not included in the instruction were excavated in this study. It underscores the potential of mining the FAERS database to uncover new ADEs, thereby addressing the gaps in drug safety data. This study highlights new ADE signals including renal and urinary disorders, as well as benign, malignant and unspecified neoplasms (including polyps and cysts), which have not received adequate attention. Renal and urinary disorders is mainly manifested as bladder spasms, it is recommended to pay more attention for symptmos such as frequent urination, urgent urination, urinary insufficiency, reduced urine volume and other symptoms, strengthen renal function monitoring, in order to timely detect ADEs. In addition, 6 strongly signaled ADEs that are included in neoplasms benign, malignant and unspecified (including cysts and polyps). Due to the characteristics of local invasion and easy metastasis of malignant tumors (Scully et al., 2012; Kim, 2021), it is difficult to determine whether tumor metastasis and tumor progression are caused by neratinib, and it is considered to be poor drug efficacy or drug resistance, rather than ADE. Additionally, skin and subcutaneous tissue disorders such as onycholysis, onychoclasis and acneiform dermatitis were identified in this study, which were not documented in the manual. Study has shown that epithelial growth factor receptor (EGFR) plays crucial role in the proliferation of the epidermis and hair follicles, which can stimulate epidermal growth and accelerate wound healing (Ehmann et al., 2011; Nanba et al., 2021; Amberg et al., 2019). Neratinib not only inhibits the phosphorylation of HER2 receptor, but also inhibits the EGFR pathway, thereby inhibiting the growth of epidermis and hair follicles, resulting in a variety of skin and hair-related adverse reactions (Hamid et al., 2019). Therefore, it is recommended to guide patients to pay attention to moisturizing the skin, avoid excessive exposure to the sun, protect nails, and avoid contact with skin irritants.

### 4.4 Limitations

Although this study is based on a large sample of real world data, however it has certain limitation. Firstly, the FAERS database relies on self reporting, which may lead to underreporting, duplicate reporting, and inaccurate reporting, potentially biasing the study results. Secondly, most of the data in FAERS originates from the United States, so the results of this study may have some deviation from the actual situation of other countries. Lastly, the signals of adverse drug events (ADEs) only represent statistical correlations, and further clinical observations and studies are necessary to establish causal relationship biologically. Taken together, this study serves as a significant exploration in signal mining. Despite its limitations, findings emphasize the need for subsequent close monitoring and necessity of further investigation through case-control studies.

## 5 Conclusion

This study provides comprehensive scientific basis for the safety assessment of neratinib through multi-level and multi-angle analysis. By employing four disproportionality methods we scrutinized and analyzed ADEs signals associated with neratinib within FAERS database. Our results unveiled a multitude of postmarketing ADEs resembling those outlined in the manual, alongside additional reports necessitating further regulatory scrutiny to assess their significance. Notably, althoughcertain ADEs, like breast cellulitis, bladder spasm, and onycholysis, which, despite occurring at lower frequencies, exhibit substantial signal strength, indicating the need for heightened attention and deeper investigation. Urgent research is vital to comprehensively understand the safety profile of neratinib, thereby enhancing its effective application in clinical practice.

## Data Availability

The original contributions presented in the study are included in the article/Supplementary Material, further inquiries can be directed to the corresponding author.
