# Supplementary material for: Neratinib safety evaluation: real-world adverse event analysis from the FAERS database
Source: Front Pharmacol. 2024 Sep 13;15:1425171. doi: 10.3389/fphar.2024.1425171 (PMC11427278; doi:10.3389/fphar.2024.1425171)
Supplement: Supplementary file 1 [file Table1.DOC]

Supplementary Table 1 ROR, PRR, BCPNN, and EBGM methods, formulas, and thresholds.

| Method | Formula | Threshold |
| --- | --- | --- |
| ROR | $ROR=\frac{ad}{bc}$ $\text{95\%CI}=\text{e}^{\text{ln(ROR)}\pm1.96\sqrt{\frac{1}{\text{a}}+\frac{1}{b}+\frac{1}{c}+\frac{1}{d}}}$ | The criteria of positive safety signal detection: the lower limit of 95% CI > 1, N ≥ 3 |
| PRR | $PRR=\frac{a(c+d)}{c(a+b)}$ $x^{2}=\frac{(a+b+c+d)(ad-bc)^{2}}{(a+b)(c+d)(a+c)(b+d)}$ | The criteria of positive safety signal detection: PRR ≥ 2, χ2 ≥4, N ≥ 3 |
| BPCNN | $IC=l\text{og2}\frac{\text{a(a}+\text{b}+\text{c}+\text{d)}}{\text{(a}+\text{c)(a}+\text{b)}}$ $95\%C\text{I}=\text{E(IC)}\pm2\times\sqrt{\text{V(IC)}}$ | The criteria of positive safety signal detection: IC025 > 0 (IC025: the lower bound of 95% CI) |
| EBGM | $EBGM=\frac{a(a+b+c+d)}{(a+c)/(a+b)}$ $95\%CI=\text{e}^{\ln(EBGM)\pm1.96\sqrt{\frac{1}{a}+\frac{1}{b}+\frac{1}{c}+\frac{1}{d}}}$ | The criteria of positive safety signal detection: EBGM05 > 2 (EBGM05: the lower bound of 95% CI) |
